# ALS-linked FUS exerts a gain of toxic function involving aberrant p38 MAPK activation

**DOI:** 10.1038/s41598-017-00091-1

**Published:** 2017-03-08

**Authors:** Reddy Ranjith K. Sama, Claudia Fallini, Rodolfo Gatto, Jeanne E. McKeon, Yuyu Song, Melissa S. Rotunno, Saul Penaranda, Izrail Abdurakhmanov, John E. Landers, Gerardo Morfini, Scott T. Brady, Daryl A. Bosco

**Affiliations:** 1 Department of Neurology, University of Massachusetts Medical School, Worcester, MA USA; 2 Department of Anatomy and Cell Biology, University of Illinois at Chicago, Chicago, IL USA; 3 Department of Genetics, School of Medicine, Yale University, New Haven, CT USA; 4 Marine Biological Laboratory, Woods Hole, MA USA

## Abstract

Mutations in Fused in Sarcoma/Translocated in Liposarcoma (FUS) cause familial forms of amyotrophic lateral sclerosis (ALS), a neurodegenerative disease characterized by progressive axonal degeneration mainly affecting motor neurons. Evidence from transgenic mouse models suggests mutant forms of FUS exert an unknown gain-of-toxic function in motor neurons, but mechanisms underlying this effect remain unknown. Towards this end, we studied the effect of wild type FUS (FUS WT) and three ALS-linked variants (G230C, R521G and R495X) on fast axonal transport (FAT), a cellular process critical for appropriate maintenance of axonal connectivity. All ALS-FUS variants impaired anterograde and retrograde FAT in squid axoplasm, whereas FUS WT had no effect. Misfolding of mutant FUS is implicated in this process, as the molecular chaperone Hsp110 mitigated these toxic effects. Interestingly, mutant FUS-induced impairment of FAT in squid axoplasm and of axonal outgrowth in mammalian primary motor neurons involved aberrant activation of the p38 MAPK pathway, as also reported for ALS-linked forms of Cu, Zn superoxide dismutase (SOD1). Accordingly, increased levels of active p38 MAPK were detected in post-mortem human ALS-FUS brain tissues. These data provide evidence for a novel gain-of-toxic function for ALS-linked FUS involving p38 MAPK activation.

## Introduction

Mutations in *Fused in Sarcoma/Translocated in Liposarcoma* (*FUS/TLS* or *FUS*) are causative for amyotrophic lateral sclerosis (ALS)^1, 2^, a progressive and fatal neurodegenerative disease characterized by progressive dying back degeneration of both upper and lower motor neurons^3^. Analysis of pathogenic mechanisms associated with mutant FUS in mammalian cells is complicated because FUS is a multifunctional protein engaged in numerous aspects of RNA and DNA processing, and overexpression of FUS WT proved cytotoxic^3^. Recent reports describing a series of transgenic FUS animal models provide strong evidence for mutant FUS exerting a gain-of-toxic function in the cytoplasm that results in motor neuron degeneration^4–6^. However, specific toxic function(s) associated with FUS mutations have not been elucidated, and could involve defects in RNA-related processes^3^, cytoskeletal transport^7^ and/or the proteostasis network^8^.

Deficits in fast axonal transport (FAT) and abnormal activation of protein kinases are early pathogenic events in both SOD1-related and sporadic forms of ALS^9–15^, but whether ALS-linked FUS affects FAT has not been evaluated. To address this issue, recombinant FUS proteins were perfused into isolated squid axoplasm and the effect on FAT examined. Although FUS WT had no discernable effect, ALS-FUS variants robustly inhibited anterograde (conventional kinesin-dependent) and modestly inhibited retrograde (cytoplasmic dynein-dependent) FAT when perfused into squid axoplasm. Irrespective of whether the disease-causing mutation affects the nuclear localization signal (e.g., R521G or R495X) or the glycine-rich domain (e.g., G230C) of FUS, all disease variants tested similarly inhibited FAT. Addition of mutant FUS with the molecular chaperone Hsp110 mitigated this impairment, suggesting that a misfolded conformation of mutant FUS underlies this gain-of-toxic function.

Pharmacological experiments further indicated that impairments in FAT in squid axoplasm and in axon outgrowth in murine primary motor neurons by mutant FUS both require activation of the p38 MAPK pathway, which has been broadly implicated in various neurodegenerative disorders including Alzheimer’s Disease and ALS^16–19^. Indeed, aberrant activation of the p38 MAPK pathway by ALS-related forms of Cu, Zn superoxide dismutase (SOD1) was previously demonstrated in squid axoplasm^10, 12, 20^ and transgenic mouse models expressing ALS-linked mutant SOD1^12, 21–23^. We detected elevated levels of phosphorylated p38 MAPK in post-mortem CNS tissues derived from individuals harboring FUS mutations, providing support that the phenotypes observed *in vitro* in this report are relevant to the human disease. Although SOD1 and FUS have no known overlapping physiological functions, mutant and misfolded forms of these proteins appear to converge on a common pathogenic pathway that involves p38 MAP kinases. Together these results identify p38 MAPK activation as a novel gain of toxic function of mutant FUS that results in reductions in FAT and strengthens the association between the p38 MAPK axis and neurodegeneration in ALS.

## Results

### ALS-FUS inhibits fast axonal transport

**Figure 1:**
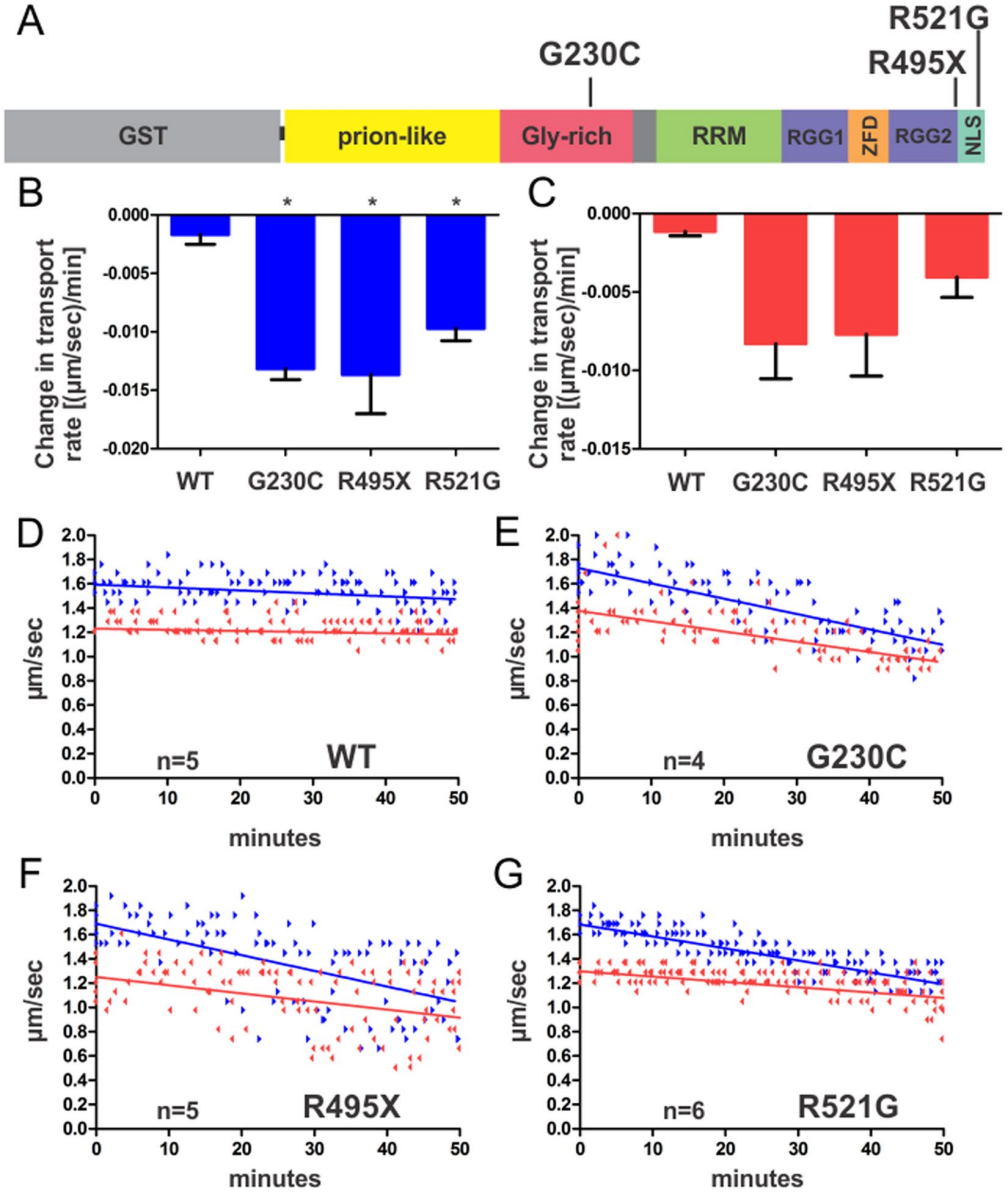
ALS-linked mutant FUS proteins impair anterograde and retrograde FAT. (**A**) ALS-linked mutations (G230C, R495X and R521G) investigated in this study are mapped onto the domain structure of GST-FUS. RRM = RNA recognition motif, RGG = arginine-glycine-glycine-rich, ZFD = zinc-finger domain and NLS = nuclear localization signal. (**B–G**) FUS proteins (2.5 μM) were perfused into isolated squid axoplasm and fast axonal transport (FAT) rates (μm/s) of membrane bounded-organelles measured as a function of time (minutes). For (**B**,**C**), slopes of the linear best fits for velocities obtained for each axoplasm (**D–G**) were averaged and plotted as bar graphs in Graphpad Prism with the standard error of the mean (SEM) for anterograde (blue bars; **A**) and retrograde (red bars; **B**) velocities. Those conditions with statistical significance (*p* < 0.05) relative to FUS WT are denoted by *, determined by one-way Anova and Tukey post-hoc test for multiple comparisons. (**D–G**) Motility plots comprised of the raw data for every axoplasm (‘n’ denotes the number of axoplasms analyzed for each condition) are shown, where linear best fits of the compiled data are shown for anterograde (blue arrowheads; linear fit shown as a solid blue line) and retrograde (red arrowheads; linear fit shown as a solid red line) directions. Protein obtained from at least two independent protein expression and purifications were included for each FUS variant.

Cytoskeletal abnormalities, including impaired axonal transport, are implicated as early events in the pathogenesis of ALS and other neurodegenerative disorders^24^. We have demonstrated the utility of isolated squid axoplasm as a powerful *ex vivo* system to study alterations in FAT induced by disease-related neuropathogenic proteins^10, 12, 20, 24–27^, which were confirmed in mammalian disease models^12^ and in human post-mortem central nervous system (CNS) tissues^10, 28, 29^. Here, we sought to determine whether ALS-linked mutant FUS proteins also affect FAT in the squid axoplasm preparation. To this end, recombinant wild-type (WT) and three distinct ALS-linked FUS variants (G230C, R521G and R495X) were expressed and purified as glutathione-S-transferase (GST)-tagged recombinant proteins (Fig. 1A). Mutations R495X and R521G are located within a nuclear localization signal (NLS) that induce FUS translocation from the nucleus to the cytoplasm^30^, with R521 representing a “hot-spot” for ALS-linked FUS mutations^31^. The R495X truncation mutation leads to an omission of the entire NLS and a more aggressive disease phenotype (i.e., earlier age of onset and shorter disease duration) in affected patients^30, 32^. Conversely, mutations in the Gly-rich region of FUS, such as G230C, do not affect the cellular localization of exogenously expressed FUS in mammalian cells^33^. Because untagged FUS is intrinsically prone to aggregation^34^ and undergoes liquid-liquid phase separation^35–38^, the GST tag was maintained throughout this study to enhance the solubility of FUS proteins. Recombinant GST-FUS has been employed in various functional assays *in vitro*, and therefore the tag is not expected to disrupt the fold or function of this protein^34, 39, 40^. Moreover, the GST protein itself does not have an effect on FAT^25^.

Next, FUS proteins were perfused into squid axoplasm and their effect on FAT determined using video-enhanced contrast differential interference (DIC) microscopy^27^. The use of DIC microscopy in the axoplasm preparation allows for quantitation of anterograde (conventional kinesin-dependent) and retrograde (cytoplasmic dynein-dependent) transport of membrane-bounded organelles (MBOs) in real time^27, 41^. Because the plasma membrane is removed from the axon in a manner that leaves intact the FAT machinery and polarity of the axonal cytoskeleton, recombinant FUS proteins could be directly perfused into the axoplasm and their effect on FAT quantitatively measured^27^. Recombinant FUS proteins were perfused into squid axoplasm at 2.5 µM, a physiologically relevant concentration in mammalian cells^38^. Perfusion of FUS WT (2.5 µM) had no effect on either anterograde or retrograde FAT over the entire time course of the assay (50 min; Fig. 1B–D). In striking contrast, perfusions with either FUS G230C, R495X or R521G significantly inhibited anterograde FAT and, to a lesser extent, retrograde FAT (Fig. 1B,C,E,F,G). Together, these data reveal FAT inhibition as a novel, toxic gain of function for mutant FUS. Given the similar effect of all three variants tested, we focused our next experiments on FUS R521G, a mutation with high frequency in the ALS-FUS population^31^.

### Inhibition of FAT by ALS-FUS is mediated by p38 MAPK

**Figure 2:**
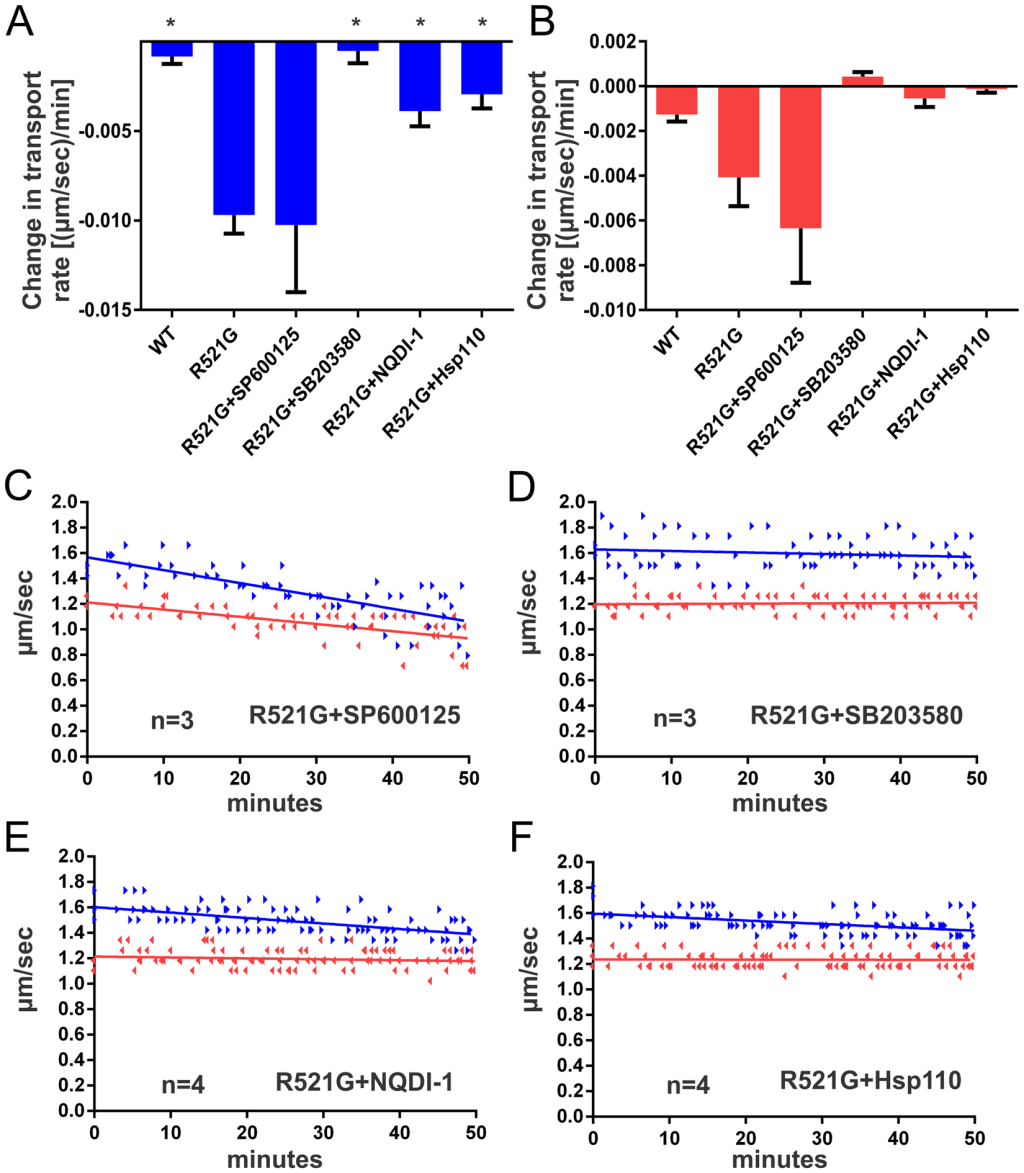
The inhibitory effect of mutant-FUS on FAT is mediated by activated p38 MAPK. FUS R521G was co-perfused with the indicated pharmacological inhibitor or Hsp110 into squid axoplasm and fast axonal transport evaluated as described in Fig. 1. (**A–C**) Co-perfusion of the pharmacological JNK kinase inhibitor SP600125 (0.5 μM) with R521G failed to rescue FAT inhibition. In contrast, inhibition of the p38 MAPK pathway by co-perfusion of either SB203850 (5 μM) or NQDI-1 (20 μM) prevented the toxic effects of FUS R521G on FAT (**A**,**B**,**D**,**E**). (**F**) Similarly, co-perfusion of Hsp110 (0.6 μM) and R521G ameliorated the inhibition of FAT by mutant FUS. Those conditions with statistical significance (*p* < 0.05) relative to FUS R521G are denoted by *, determined by one-way Anova and Tukey post-hoc test for multiple comparisons.

The inhibitory effect of mutant FUS proteins on FAT was independent from nucleus-dependent activities (i.e., transcription) because neuronal cell bodies are absent from the axoplasm preparation^41^. Endogenous axoplasmic kinases and phosphatases are plausible candidates, as kinases mediate the inhibitory effect of other neuropathogenic proteins on FAT by promoting abnormal phosphorylation of conventional kinesin and cytoplasmic dynein, major molecular motors responsible for FAT in mature neurons. For example, activation of cJun N-terminal kinases (JNK) was found to mediate the inhibitory effect of pathogenic androgen receptor and huntingtin on FAT^25, 26^. Based on these precedents, we co-perfused the JNK-specific kinase inhibitor SP600125 with FUS R521G. However, inhibition of JNK did not rescue the inhibitory effect of mutant-FUS on FAT (Fig. 2A–C).

The beta-isoform of p38 mitogen-activated protein kinase (MAPK) also inhibits both anterograde and retrograde FAT in isolated squid axoplasm^12^, and ALS-related forms of ALS have been shown to inhibit FAT through a mechanism involving p38 activation^10, 12, 20^, prompting us to evaluate the potential role of this kinase. Strikingly, co-perfusion of FUS R521G with the p38 MAPK pharmacological inhibitor SB203580 completely prevented the adverse effects of this protein on FAT, restoring FAT rate values to that of control axoplasms (Fig. 2A,B,D). p38 kinases are typically activated by sequential activation of upstream MAPK kinases (MKKs) and MAPK kinase kinases (MKKKs). An example of the latter is apoptosis signal-regulating kinases (ASKs), which mediates the inhibitory effects of pathogenic SOD1 variants on FAT^20^. Therefore, we assessed the effect of a pharmacological ASK1 inhibitor (NQDI-1) on FUS-mediated inhibition of FAT. Significantly, NQDI-1 also prevented the adverse effects of FUS R521G on FAT (Fig. 2A,B,E). Taken together, results from vesicle motility assays indicate that pathogenic forms of two seemingly different ALS-linked proteins (FUS and SOD1) converge on a common pathway that involves aberrant activation of p38-specific MAP kinase pathways. There are, however, mechanistic differences between SOD1- and FUS-induced inhibition of FAT. Whereas both anterograde and retrograde FAT are impaired by mutant FUS (Figs 1 and 2), only anterograde FAT was significantly inhibited by misfolded SOD1^10, 12, 20^. We previously detected phosphorylated p38 MAPK in squid axoplasm perfused with misfolded SOD1 by immunoblot analysis^12^. However, the same mammalian anti-p38 MAPK antibody used previously failed to detect activated p38 MAPK in squid perfused with mutant FUS (data not shown). These results suggest that different p38 MAPK isoforms may be activated by mutant FUS compared to misfolded SOD1, and that these different isoforms may not be detected by our available anti-phosphorylated p38 antibodies.

### The molecular chaperone Hsp110 mitigates the effect of mutant FUS on FAT

Protein misfolding is a hallmark feature of neurodegenerative diseases, including ALS^8^. Indeed, FUS aggregates are detected within neurons and glia of human post-mortem ALS-FUS tissue^2, 42^, in cultured cells^1^, and *in vitro* ^34^. To address the possibility that a misfolded form of mutant FUS activates the p38 MAPK pathway, the molecular chaperone Hsp110 was co-perfused with mutant FUS protein. Hsp110 has been shown to stabilize unfolded proteins and synergize with other chaperones such as Hsp(c)70 and Hsp40 to disaggregate protein assemblies *in vitro* ^43, 44^. Specifically, Hsp110 prevented mutant SOD1 from activating p38 MAPK in axoplasm^20^ and overexpression of Hsp110 extended the survival of mutant SOD1 transgenic mice^45^. Co-perfusion of substoichiometric amounts of Hsp110 to FUS R521G (1:4) significantly attenuated the inhibitory effect of mutant-FUS on both anterograde and retrograde FAT rates, which were similar to those observed in FUS WT-perfused axoplasms (Fig. 2A,B,F). These data suggest that a misfolded conformation of mutant-FUS may underlie its inhibitory effect on FAT^20^.

**Figure 3:**
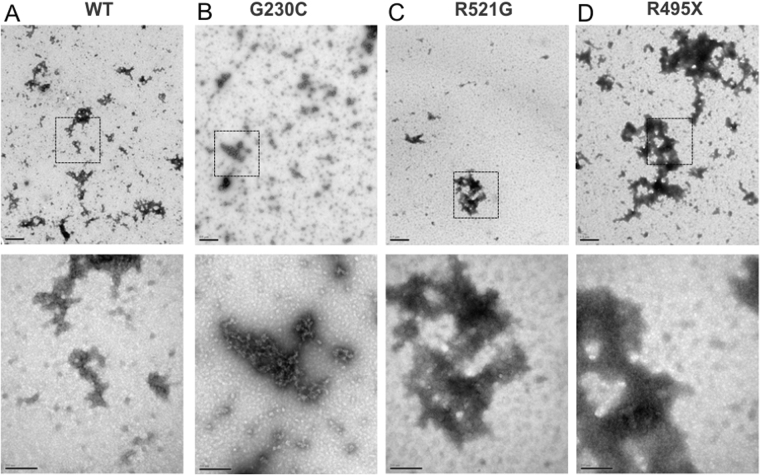
The morphology of FUS species assessed by electron microscopy. Representative electron microscopy (EM) images of negatively stained FUS WT (**A**), G230C (**B**), R521G (**C**) and R495X (**D**) proteins. Low magnification images are shown in the top row (scale bar represents 0.5 μm). High magnification images are shown for insets (dashed squares) in the bottom row (scale bar represents 200 nm). FUS exhibits heterogeneous morphologies across all samples.

Next, we attempted to identify the putative misfolded mutant FUS species. Recombinant FUS protein stocks used for squid axoplasm studies were analyzed using electron microscopy (EM). There was no visible protein precipitate, nor were any proteins in a hydrogel-like state. Instead, EM images revealed a mixture of oligomeric and higher-ordered protein assemblies for both FUS WT (Fig. 3A) and mutant FUS proteins (Fig. 3B–D). This morphological heterogeneity precluded the identification of a specific mutant FUS species that would trigger the defects in FAT.

### Inhibition of p38 MAPK rescues impaired axon outgrowth in mammalian motor neurons expressing mutant FUS

**Figure 4:**
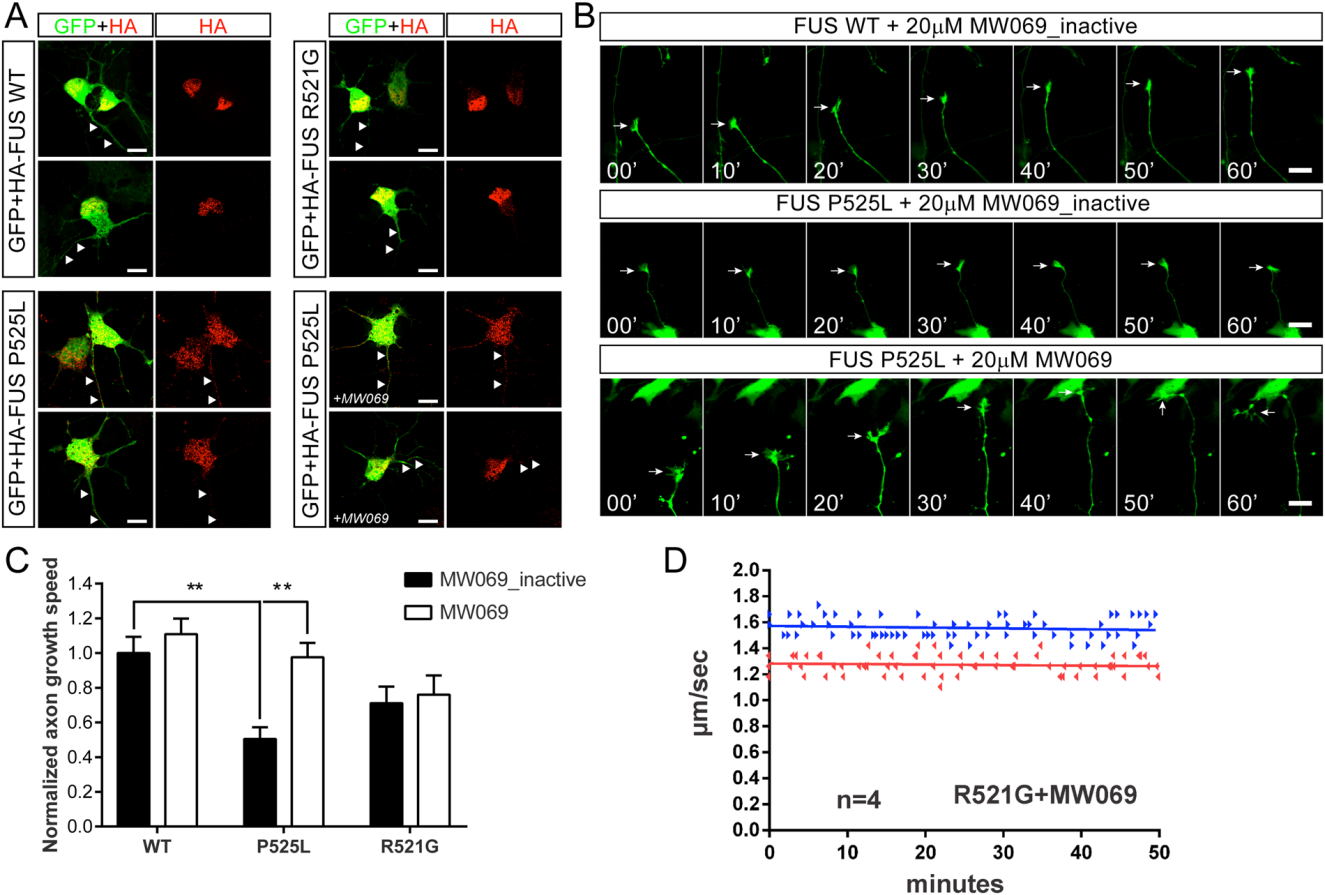
Mutant FUS impairs axon outgrowth in motor neurons through a mechanism involving p38 MAPK activity. Murine motor neurons were transiently co-transfected with FLAGHA-FUS WT, R521G or P525L and green fluorescent protein (GFP) at 2 days *in vitro* (DIV). Motor neurons were cultured in 20 μM MW069, a potent and selective p38 MAPK inhibitor, or an inactive analogue, MW069_inactive. (**A**) FUS WT and R521G are predominately expressed within the nucleus of motor neurons, where as FUS P525L is expressed in the nucleus and within axons (white arrowheads). FUS P525L localization is similar whether motor neurons are treated with MW069_inactive or MW069 (+MW069). (**B**) Montages corresponding to live cell imaging of axon outgrowth (white arrow) over a 60 min time course for the indicated condition. Note that the growth cone of a FUS P525L expressing motor neuron is stalled in the presence of MW069_inactive (middle panel) relative to the active form of MW069 (bottom panel). See Supplemental Video S1 for live cell imaging of all conditions. (**C**) Quantification of axon outgrowth speed compiled from n = 3 biological experiments normalized to the WT + MW069_inactive condition. Statistical significance (**p < 0.01) of pertinent comparisons are indicated. Additional significant comparisons include FUS WT + MW069 versus FUS R521G + MW069_inactive (p < 0.05) and FUS WT + MW069 versus FUS P525L + MW069_inactive (p < 0.0001). (**D**) The inhibition of FAT by FUS R521G is blocked by MW069 in squid axoplasm. The motility plot for FUS R521G in the absence of MW069 is shown in Fig. 1G.

Axonal defects have been reported in cortical neurons ectopically expressing mutant FUS^46^, and endogenous expression of mutant FUS in motor neurons derived from patient iPSCs recapitulated ALS-relevant phenotypes such as hyperexcitability^47^. Here, we sought to determine whether mutant FUS-induced toxicity within mammalian motor neurons involves p38 MAPK activity as demonstrated for squid axoplasm (Figs 1 and 2). To this end, murine motor neurons were transiently transfected at day *in vitro* (DIV) 2 with FLAG/HA-FUS WT, R521G and P525L in the presence of either MW069 (20 μM), an inhibitor with higher selectivity for p38 MAPK than SB203580, or a structurally related, inactive analogue of MW069 (MW069_inactive, 20 μM) as a negative control^48^. Motor neurons were co-transfected with the green fluorescent protein (GFP) to facilitate visualization of axonal outgrowth using live cell imaging (Fig. 4A and Supplemental Video S1). In the presence of MW069_inactive, axon outgrowth was significantly impaired in motor neurons expressing FUS P525L compared to those expressing FUS WT (Fig. 4B,C). Strikingly, axonal outgrowth rates in FUS P525L expressing motor neurons were restored to WT FUS levels in the presence of the active MW069 inhibitor (Fig. 4B,C). Axon outgrowth speed was also reduced in FUS R521G expressing motor neurons treated with MW069_inactive, although this reduction was not statistically significant compared to FUS WT with MW069_inactive treatment (p = 0.32). Moreover, the active MW069 inhibitor had no effect on axon outgrowth speed in FUS R521G expressing motor neurons (Fig. 4C). These latter results are suggestive of a toxic function of FUS R521G within the nucleus, where this protein is predominately expressed within intact motor neurons, that is unrelated to p38 MAPK activation (Fig. 4A). Supporting this idea, FUS P525L is severely mislocalized to the cytoplasm^33^ and expressed at detectable levels within the axon (Fig. 4A). Analogous to the direct perfusion of mutant FUS proteins into squid axoplasm (Figs 1 and 2), the presence of mutant FUS within axons of intact motor neurons was sufficient to induce toxicity through a mechanism involving p38 MAPK activity. Importantly, the MW069 inhibitor also rescues the inhibition of FAT by mutant FUS in squid axoplasm (Fig. 4D).

### Increased activation of p38 MAPK is detected in human post-mortem ALS tissue

**Figure 5:**
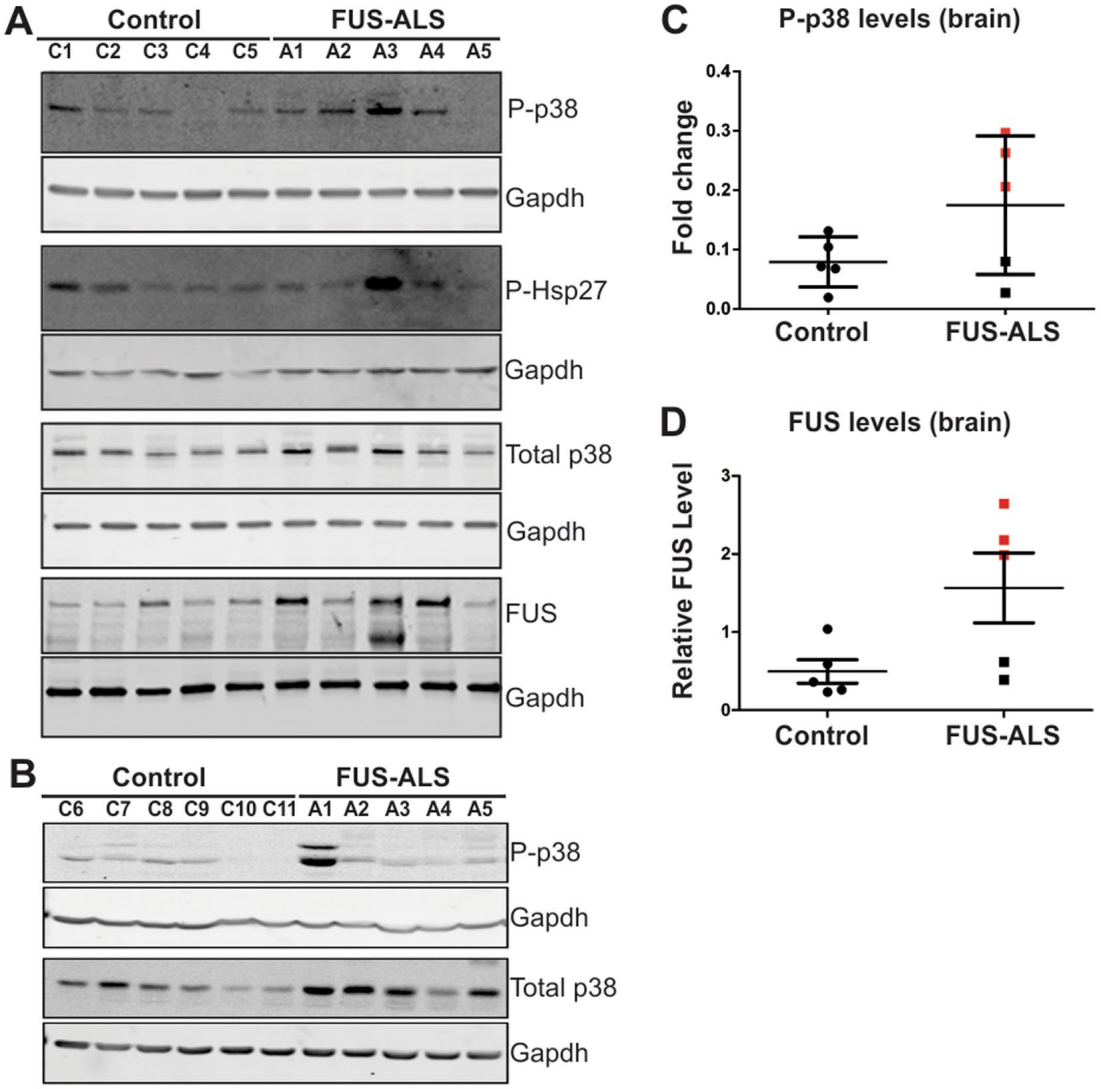
Increased levels of phosphorylated p38 MAPK in human post-mortem CNS tissues derived from individuals with ALS-FUS. Immunoblot analysis of frozen post-mortem brain (**A**) and spinal cord (**B**) tissues derived from non-disease control (C1-C11) and ALS individuals harboring *FUS* mutations (A1-R521G; A2-H517Q; A3- ex14del; A4-P525L and A5- bp1408del) with the indicated antibodies (see Supplemental Table S2 for detailed patient information). (**C**) Quantification of phosphorylated (active) p38 (p-p38) by immunoblot analysis revealed higher levels of p-p38 MAPK in ALS cases (cases with levels above the control mean are indicated by red squares), compared to control cases. P-p38 values were normalized to total p38 levels. (**D**) Quantification of FUS levels in brain normalized to Gapdh. ALS cases with FUS levels above the control mean are indicated by red squares. (**C**,**D**) Although, there appears to be a trend in the data, differences between ALS cases and controls did not reach statistical significance due to the small sample size and inherent variability of human postmortem samples.

To evaluate the disease relevance of findings in the squid axoplasm preparation, we investigated the relationship between mutant FUS expression and p38 MAPK activation in nerve tissue samples obtained from ALS-FUS cases. Specifically, we examined post-mortem CNS tissues derived from ALS individuals harboring different FUS mutations (Supplemental Table S2), including R521G, H517Q, a splice site mutation (ex14del) that causes skipping of exon14, P525L and a base pair deletion (bp1408del) (A1-A5, respectively, in Fig. 5). Both ex14del and bp1408del are predicted to cause a frame shift, resulting in FUS proteins comprised of 477 and 526 amino acids, respectively, but without the correct FUS sequence at the C-terminus. All cases exhibited both upper and lower motor neuron pathology at autopsy (Supplemental Table S2), consistent with FUS pathology reported in both motor cortex and spinal cord (SpC) tissues^42^.

Next, we evaluated p38 MAPK activation in post-mortem brain and SpC tissues by immunoblotting using an antibody that selectively recognizes catalytically active forms of p38 (p-p38) phosphorylated by upstream MKKs. A phosphorylation-independent antibody against p38 MAPK (total p38) served as internal control for expression and loading. An anti-GAPDH antibody served as an additional loading control for each analysis. Notably, when compared to control tissues there were elevated levels of p-p38 relative to total p38 MAPK in both ALS motor cortex (Fig. 5A) and SpC (Fig. 5B) tissues, with a more pronounced effect in brain samples than in SpC. Three out of five ALS cases (A2-4) expressed elevated levels of p-p38 in tissue derived from the motor cortex (Fig. 5A), with p-p38 levels above the mean of the control set (Fig. 5C). Additional immunoblot analyses were performed on the brain samples. For example, a qualitative direct correlation between p-p38 and p-Hsp27, a target of p-p38^49^, provided independent evidence of increased p38 activation in these samples (Fig. 5A). Interestingly, three of the five ALS cases (A1, A3 and A4), all expressing mutant forms of FUS that are expected to exhibit cytoplasmic mislocalization^1, 30, 33^, express relatively high levels of FUS (Fig. 5A,D). This trend is consistent with the notion that mislocalized FUS fails to properly autoregulate its expression^50^. Two FUS bands were detected in the immunoblot for A3, the ALS case with the highest FUS levels; the faster migrating band is attributed to the mutant (477 amino acids) form, whereas the slower migrating protein (526 amino acids) is produced from the wild-type allele (Fig. 5A). The differences between control and ALS did not reach statistical significance for p-p38 (Fig. 5C; p = 0.16) or FUS (Fig. 5D; p = 0.053) biochemical quantification, likely due to the relatively small number of human cases that were available for this study and the inherent variability of human postmortem tissue, but the trend was consistent with increased p-p38 levels.

**Figure 6:**
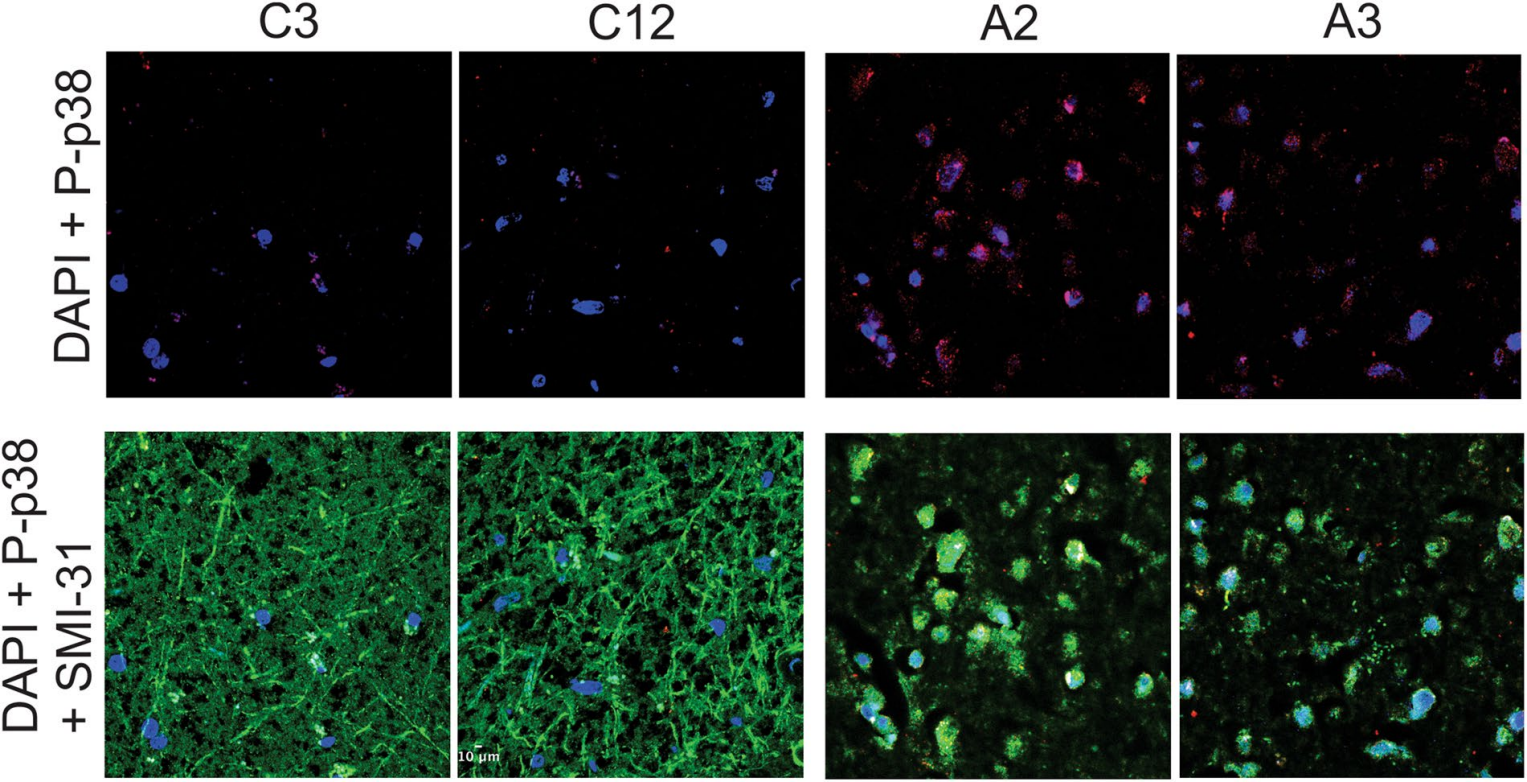
Active p38 MAPK in neurons within post-mortem motor cortex tissues from individuals diagnosed with ALS-FUS. Brain sections from paraffin-embedded tissue samples obtained from two control (C3 and C12) and two ALS-FUS (A2 and A3) cases were probed with antibodies against phosphorylated, catalytically active p38 (P-p38; red), phosphorylated neurofilament H (SMI31; green) as a marker of neuroaxonal integrity, and the nuclear stain DAPI (blue). Scale bar represents 10 μm. Low magnification images are shown in Supplemental Figure S3 **.** The P-p38 signal is higher, and SMI31 signal lower, in both ALS cases compared to controls; see Supplemental Figure S4 for quantification.

Paraffin-fixed motor cortex tissue was available for a subset of our ALS (A2 and A3) and control (C3 and C12) cases that could be used for immunostaining. Consistent with results from the immunoblot analysis, immunostaining of motor cortex tissue revealed higher p-p38 immunoreactivity from ALS cases, compared to control cases (Fig. 6). Tissues were co-stained with SMI31, an antibody reactive for phosphorylated neurofilament H that can be used to assess neuroaxonal integrity^51^. As expected from the marked degeneration of neurites that characterizes ALS pathology^52^, SMI31 reactivity was markedly reduced in both ALS cases compared to controls (Fig. 6 and Supplemental Figure S3). Quantification of the ratio of p-p38 to SMI31 revealed a statistically significant difference between controls (0.044 +/− 0.010) and ALS (0.243 +/− 0.017, p < 0.0001; Supplemental Figure S4). Elevated levels of phosphorylated p38 MAPK in affected ALS-FUS tissues shown here are consistent with reports of elevated p38 MAPK in SOD1^12, 22, 53^ and sporadic^16^ forms of ALS.

## Discussion

**Figure 7:**
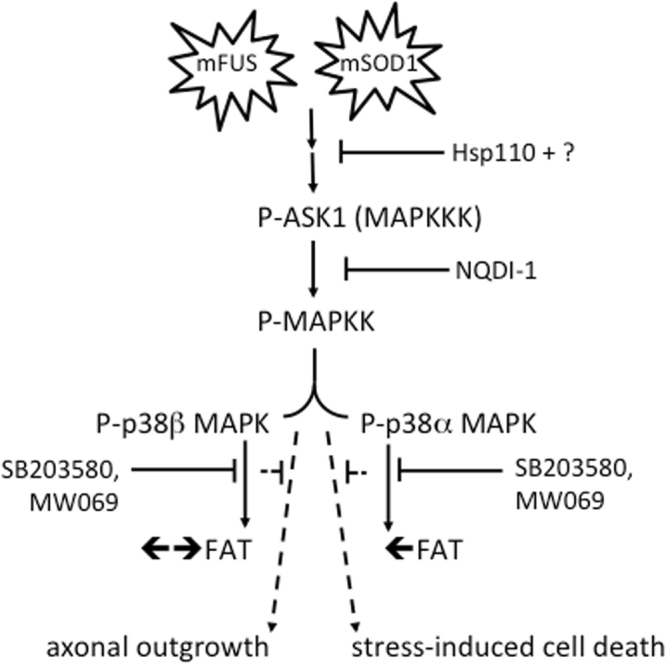
Model for aberrant activation of p38 MAP kinase(s) by mutant and misfolded ALS-associated proteins. Available data (from this study and others^10, 12, 20^) support a model whereby mutant and misfolded forms of FUS (mFUS; left) and SOD1 (mSOD1; right) induce the aberrant activation of the p38 MAPK pathway. Hsp110 likely synergizes with other chaperones to ameliorate the effects of mSOD1 and mFUS, possibly upstream of ASK1 and additional unidentified factors. While mSOD1 and mFUS converge on p38 MAPK activation, perfusion of these proteins into squid axoplasm have differential effects on FAT; mFUS inhibits both anterograde (←) and retrograde (→) FAT (Figs 1 and 2) whereas mSOD1 only inhibits anterograde FAT^10, 12, 20^, consistent with inhibition of the beta and alpha isoforms of P38 MAPK, respectively^12^. In addition to FAT inhibition in squid (solid lines), mFUS- and mSOD1-induced activation p38 MAPK can manifest different phenotypes in mammalian systems (dashed lines), including but not limited to inhibition of axon outgrowth (Fig. 4) and enhanced susceptibility to cell stress^12^. This figure is adapted from Song *et al.* ^20^.

The identification of mutant FUS-specific phenotypes have been elusive, in part because FUS WT overexpression elicits a toxic phenotype in many model organisms^34, 54–56^. Important insights into the mechanism of FUS-mediated pathogenesis were recently revealed by two independent studies, which compared FUS knockout mice to a series of transgenic mouse lines expressing the human FUS transgene at or below endogenous FUS levels^4, 5^. Both studies demonstrated that a loss of FUS expression does not cause motor neuron degeneration, whereas cytoplasmic mislocalization of FUS due to deletions or point mutations within the nuclear localization signal induced age-dependent, motor neuron degeneration^4, 5^. Collectively, these *in vivo* studies strongly implicated a toxic gain of function mechanism in FUS-mediated ALS pathogenesis. However, specific toxic effects of mutant FUS in the cytoplasm remained unknown. The present study using isolated squid axoplasm sheds new light on this question, as we demonstrate that mutant, but not WT forms of FUS, impair FAT through a mechanism dependent on activation of p38 MAPK (Fig. 7).

Axonal transport defects are a common pathological feature of neurodegenerative disorders^24^, and represent an early, pre-symptomatic event in motor neuron diseases including familial and sporadic forms of ALS^24, 57^. Evidence in humans and ALS transgenic model organisms support a ‘dying back’ pattern for motor neuron death that is initiated by pathogenic events within the distal axon, including the breakdown of neuromuscular junctions (NMJs)^11, 57^. Consistent with a common disease phenotype associated with different genetic causes of ALS, dysfunctional NMJs preceded the loss of neuronal cell bodies in mutant FUS mice^5^. Transient expression of the cytoplasmically mislocalized FUS P525L variant resulted in reduced axon outgrowth in primary motor neurons (Fig. 4), consistent with observations of abnormal axonal morphologies in primary cortical neurons expressing mutant FUS^46^. In addition, recent work using *Drosophila* larval motor neurons reported a reduction in anterograde transport of MBOs induced by both WT and the cytoplasmically mislocalized P525L mutant FUS variant^58^. While physiologically relevant concentrations of FUS WT did not affect FAT in isolated axoplasm (Figs 1 and 2), the *Drosophila* study involved overexpression of FUS, which in some cases can cause toxicity^34, 54–56^. The effects of FUS WT on FAT in *Drosophila* may also represent an epiphenomenon of FUS-modulated changes in gene expression or other nuclear activities^3, 59^. Supporting this view, a trend toward reduced axon outgrowth speed was observed in motor neurons expressing the predominately nuclear FUS R521G variant (Fig. 4), but this effect was not mitigated by p38 MAPK inhibition as observed for the predominantly cytoplasmic FUS P525L mutant variant (Fig. 4). Additional work is needed to evaluate the extent to which nuclear dysfunction-specific mechanisms of FUS contribute to FAT alterations and impaired axon outgrowth within intact neurons.

Unlike other models, isolated squid axoplasm affords a unique, *ex vivo* experimental system to directly compare the effects of FUS WT and ALS-linked variants because proteins are perfused directly into axoplasm at the same concentrations. Regardless of whether these variants are predominately expressed in the nucleus (G203C), cytoplasm (R495X), or somewhere in between (R521G) within intact cells, they all inhibited FAT to a similar extent, implicating a common toxic feature among FUS mutants. The protective effect of Hsp110 (Fig. 2) further raised the possibility that a misfolded conformation underlies the toxic effect of mutant FUS on FAT. Hsp110 is nucleotide exchange factor for Hsp(c)70, and together with Hsp40 these chaperones comprise a mammalian disaggregase system that dissolves amorphous protein aggregates *in vitro* ^43, 44^. FUS variants are known to aggregate *in vitro* and *in vivo* ^34^, although FUS aggregation is not required to induce motor neuron degeneration *in vivo* ^4, 5^. Interestingly, FUS has been shown to undergo liquid-liquid phase separation and assemble into a hydrogel^35–38^. Here, all FUS samples were soluble (i.e., not visibly precipitated or in a hydrogel-like state) before they were perfused into squid axoplasm or subjected to EM. Nonetheless, EM analysis revealed the presence of soluble, self-associated FUS species in both FUS WT and mutant FUS samples (Fig. 3), precluding our ability to assign the ‘toxic’ FUS species to a particular size or morphology of FUS aggregate, as reported for other neuropathogenic proteins^60^. Our studies do not exclude the possibility that mutant FUS proteins acquire a toxic conformation within the squid axoplasm, but this possibility is difficult to investigate with current methods. Further, recombinant FUS is largely disordered, precluding structural analyses of the isolated full-length protein^3^. Thus, conformational properties of mutant FUS that confer toxicity await further study.

Inhibition of FAT by mutant FUS occurs through a mechanism that involves activation of the p38 MAPK pathway (Fig. 2). Active p38 MAPK was previously shown to phosphorylate and inhibit kinesin-1 subunits of conventional kinesin, providing a mechanistic basis by which p38 MAPK activation impairs FAT^12^. FUS overexpression was recently correlated with glycogen synthase kinase 3β (GSK-3β) kinase activation, which also affects anterograde FAT^61^, although this effect was not mutant-specific^62^. Nonetheless, these observations support the notion that disease-causing neuropathogenic proteins may exert toxicity through inappropriate activation of kinase-signaling cascades. Although our studies evaluate axon-autonomous effects, mutant forms of FUS and SOD1 would likely affect other p38 MAPK-related processes within intact mammalian neurons *in vivo*, including but not limited to transcription and activation of apoptotic pathways (Fig. 7). Intriguingly, ALS-linked forms of SOD1, a functionally and structurally dissimilar protein compared to FUS, similarly impaired FAT in squid axoplasm through activation of a p38 MAPK pathway^10, 12, 20^. In fact, multiple groups^12, 21–23^ have reported increased levels of active p38 MAPK in the SOD1-G93A transgenic ALS mouse model, as well as in human sporadic and familial ALS forms^16, 18^. Here, we detected elevated levels of phosphorylated p38 MAPK in the motor cortex of human ALS-FUS cases (Figs 5 and 6). Increased levels of active p38 MAPK in disease models of different ALS variants suggests that specific molecular components within the p38 MAPK axis may represent viable therapeutic targets to treat ALS^19^. Indeed, inhibition of p38 MAPK activity using poorly penetrant inhibitors correlates with improved motor neuron survival in ALS-SOD1 transgenic mice^22, 53^. The development of highly specific, brain-penetrant MAPK inhibitors should facilitate a better evaluation of p38 MAPK contributions to ALS-related phenotyopes *in vivo* ^19^. Collectively, findings in this work provide a novel conceptual framework for the development of therapeutic strategies aimed to prevent the adverse consequences of p38 MAPK activation in ALS and other neurodegenerative disorders.

## Materials and Methods

### Recombinant FUS expression and purification

FUS WT, R521G, R495X or G230C constructs were cloned into pGEX-6P1 vector (GE Life Sciences) and expressed in Rosetta DE3 cells (Novagen) as follows. Single bacterial colonies were inoculated into 5 ml growth medium (luria broth with 100 µg/ml ampicillin and 34 µg/ml chloramphenicol) and incubated at 37 °C while shaking for 8 h, after which these cultures were used to inoculate 150 ml growth medium (intermediate culture) that was then incubated at 30 °C for ~24 h. The intermediate cultures were used to inoculate 2L growth medium to an OD_600_ of 0.1–0.2. Cultures were grown at 20 °C until an OD_600_ ~0.8, at which time IPTG (Isopropyl β-D-1-thiogalactopyranoside) and ZnCl_2_ were added to a final concentration of 150 µM and 50 µM, respectively, and cells were grown for an additional 22 h at 20 °C. Cells were harvested by centrifugation and cell pellets were resuspended in lysis buffer (50 mM Tris pH 8.0 with 1 mM Dithiothreitol (DTT), 0.1 mM Ethylenediaminetetraacetic acid (EDTA) and protease inhibitor cocktail (Roche)). Cells were lysed by sonication on ice in the presence of 10 µg/ml RNase A. GST-fusion proteins were purified from cleared lysates with Glutathione-Agarose (Sigma, #G4510) resin according to the manufacturer’s instructions and stored at −80 °C.

### Squid axoplasm vesicle motility assays

Vesicle motility assays were performed as described previously^12, 20, 27^. GST-FUS proteins alone or in combination with either pharmacological inhibitors or Hsp110 protein were employed at the following final concentrations: 2.5 µM GST-FUS proteins, 5 µM of SB203580 (EMD Millipore; 55-938-91MG), 0.5 µM of SP600125 (EMD Millipore; 42-011-95M), 10 µM of MW-069 (a gift from Dr. M. Watterson, Northwestern University), 20 µM NQDI1 (R&D Systems; 4429) and 0.6 µM HSP110 (a gift from Dr. A. Horwich, Yale University). FAT rates shown in Figs 1 and 2 were plotted using Prism (GraphPad software). The slope of the linear best-fit line for each individual axoplasm was calculated and the mean of slopes for each condition was plotted as bar graphs using Prism.

### Transmission Electron Microscopy

Samples (10 µl) containing purified 12.5 µM GST-FUS protein was adsorbed onto a carbon stabilized, Formvar support film applied to a 200 mesh copper grid for 30 seconds. The grids were then washed with water and negatively stained with 1% aqueous uranyl acetate in water as described^63^. The stained grids were analyzed using a Philips CM10 transmission electron microscope and images were captured at both low (20,500X) and high (87,000X) magnification using a Gatan Erlangshen 785 CCD digital camera system.

### Axon outgrowth measurements in primary motor neurons

Motor neurons were isolated from embryonic (E)12.5 mouse embryos and cultured as described^64^. FLAGHA-FUS expression cassette vectors were generated from pFRT-TO-DEST-FLAGHA-FUS-WT, which was obtained from Addgene (#26373). ALS-linked mutations were introduced with the QuikChange II Mutagenesis kit (Stratagene; 200523) according to the manufacturer’s instructions. Cells were transfected at 2 days *in vitro* (DIV)^65^ with green fluorescent protein (GFP) and FLAGHA-tagged FUS constructs in a 1:2 ratio with NeuroMag (OZ Biosciences) as previously described^65^. FLAGHA-FUS plasmids were generated from pFRT-TO-DEST-FLAGHA-FUS-WT obtained from Addgene (#26373). The QuikChange II Mutagenesis kit (Stratagene; 200523) was used to introduce the R521G and P525L mutations according to the manufacture’s instructions. Twenty µM MW069 or its inactive form (MW069_inactive) were added to the culture medium after transfection to inhibit p38 activity^48^ and maintained throughout the experiment for a total of ~18–24 hours. Cells were imaged at 3 days *in vitro* (DIV) using a Nikon TiE widefield microscope equipped with temperature- and CO_2_-controlled environmental chamber. Neurobasal medium was replaced with Hybernate E Low Fluorescence (BrainBits) 1 hour prior to imaging to reduce autofluorescence and light toxicity. GFP was used to identify transfected cells. Movies were acquired with a 20x lens at a rate of 1 frame every 10 minutes for 1 hour. The speed of axon outgrowth was measured using the ImageJ plugin MTrackJ^66, 67^. Data from three independent, biological replicates were normalized to the condition with FUS WT in the presence of MW069_inactive. Statistical analysis (2-way ANOVA followed by Tukey’s multiple comparisons test) was performed using Graph Pad Prism 6 to establish statistical differences (p < 0.05) between conditions. To assess FLAGHA-FUS expression, cells were post-fixed in 4% paraformaldehyde for 15 minutes and stained with an anti-HA antibody (Cell Signaling - C29F4; 1:500) overnight at 4 °C. Secondary antibody (donkey anti-Rabbit conjugated with Alexa594; Jackson ImmunoResearch) was incubated at room temperature for 1 hour.

### Processing of post-mortem tissues

Frozen post-mortem tissues were a generous gift from Dr. Robert H. Brown, Jr. (University of Massachusetts Medical School). Frozen tissue samples (~100 mg) were transferred to 2 mL borosilicate glass tube (Wheaton, #358028) containing lysis buffer (50 mM Tris–HCl (pH 7.5) supplemented with 0.5 M NaCl, 1% NP-40, 1% deoxycholic acid, 0.1% SDS, 2 mM EDTA, protease and phosphatase inhibitors (Roche)). Tissues were homogenized at 4 °C using a motorized tissue grinder with motor (Wheaton,) and the resulting lysates cleared by centrifugation prior to total protein determination using a bicinchonic acid (BCA) based colorimetric assay (ThermoScientific). Thirty micrograms of total protein was loaded onto gels for SDS-PAGE and immunoblot analyses. The following antibodies were employed: anti-phospho-p38 MAPK (Cell Signaling, #9215), anti-total-p38 MAPK (Sigma, #M0800) and anti-GAPDH (Sigma, #G8795). Densitometry was performed using the Odyssey infrared imaging systems software (LI-COR).

Paraffin embedded tissues were acquired through the Alzheimer’s Disease Research Center at the Massachusetts General Hospital (Charlestown, MA). Paraffin was removed using progressive dilutions of alcohol and Xylene as described^68^. Tissue sections were permeabilized with 0.2% Triton-X-100 in TBS and blocked with TBS plus 2.5% goat serum (Sigma-Aldrich, G9023). Primary antibodies used were anti-phospho p38 MAPK (P-p38) (Cell Signaling CS#4511; 1:200) and SMI-31 (Covance SMI-31R, 1:1000). Secondary antibodies were goat anti-rabbit Alexa 647 serum (Invitrogen, A21244) and goat anti-mouse Alexa 488 serum (Invitrogen, A21235), both used at a 1:1000 dilution. Slides were dried and mounted using Dapi Fluoromont-G (SoutherBiotech) hard mounting media. Images were acquired using an LSM-710 confocal microscope (Zeiss). For quantitative immunofluorescence analysis, images corresponding to five regions of interest (ROIs) per case were acquired from motor cortex using the same settings. Ten images (2 cases × 5 ROIs) gathered from each channel (647 nm for P-p38 and 488 nm for SMI-31 antibodies, respectively) were processed and average pixel counts determined using standard auto-threshold algorithms embedded in ImageJ software (http://imagej.nih.gov/ij/). Statistical analysis (unpaired student t -test) with Graph Pad Prism 6 (La Jolla, CA) was used to establish statistical differences (p < 0.05) between control versus ALS-FUS cases. All procedures were repeated twice with similar results.

## Electronic supplementary material

supplementary information

Supplemental Video S1
