# Supplementary material for: ALS-linked FUS exerts a gain of toxic function involving aberrant p38 MAPK activation
Source: Sci Rep. 2017 Mar 8;7:115. doi: 10.1038/s41598-017-00091-1 (PMC5428330; doi:10.1038/s41598-017-00091-1)
Supplement: Supplementary file 1 — supplementary information [file 41598_2017_91_MOESM1_ESM.pdf]

ALS-linked FUS exerts a gain of toxic function involving aberrant p38 MAPK activation

Reddy Ranjith K. Sama[1*], Claudia Fallini[1*], Rodolfo Gatto[2*], Jeanne E. McKeon[1], Yuyu Song[3], Melissa S. Rotunno[1], Saul Penaranda[4], Izrail Abdurakhmanov[4], John E. Landers[1], Gerardo Morfini[2,4#], Scott T. Brady[2,4#], Daryl A. Bosco[1#]

[1]Department of Neurology, University of Massachusetts Medical School, Worcester MA, USA.
[2]Department of Anatomy and Cell Biology, University of Illinois at Chicago, Chicago, IL, USA.
[3]Department of Genetics, School of Medicine, Yale University, New Haven, CT, USA
[4]Marine Biological Laboratory, Woods Hole, MA, USA.

Supplemental Video S1. Live cell imaging of motor neurons expressing FUS proteins. Murine motor neurons transiently co-transfected with FLAGHA-FUS WT, R521G or P525L and green fluorescent protein (GFP) at 2 days in vitro (DIV) in the presence of either 20 μM MW069, a potent and selective p38 MAPK inhibitor, or an inactive analogue, MW069_inactive. The growth of a single axon is observed under each condition.

Supplemental Table S2. Demographic and disease information of human tissue samples.

Sample ID	Gender	Ethnicity	Age (First Symptom)	Age at death	Site of onset	FUS Mutation	sALS/fALS	Final Diagnosis-El Escorial	Tissue source	[14]CNS region	Time to freeze
C1	[1]M	[3]W	[5]N/A	65	N/A	N/A	N/A	N/A	frozen	frontal lobe	Unkown
C2	M	W	N/A	66	N/A	N/A		N/A	frozen	frontal cortex	17
C3	M	W	N/A	34	N/A	N/A	N/A	N/A	frozen	motor cortex	11
C4	[2]F	W	N/A	57	N/A	N/A	N/A	N/A	frozen	motor cortex	13
C5	F	W	N/A	73	N/A	N/A	N/A	N/A	frozen	motor cortex	20
C6	F	[4]DN	N/A	76	N/A	N/A	N/A	N/A	frozen	cord	DN
C7	F	W	N/A	42		N/A	N/A	N/A	frozen	lumbar cord	DN
C8	M	W	N/A	70	N/A	N/A	N/A	N/A	frozen	lumbar cord	<24
C9	M	W	N/A	93	N/A	N/A	N/A	N/A	frozen	lumbar cord	24
C10	M	W	N/A	52	N/A	N/A	N/A	N/A	frozen	lumbar cord	<24
C11	F	W	N/A	76	N/A	N/A	N/A	N/A	frozen	lumbar cord	<24
C12	M	W	N/A	47	N/A	N/A	N/A	N/A	Paraffin	motor cortex	3
C13	M	W	N/A	63	N/A	N/A	N/A	N/A	Paraffin	lumbar cord	16
A1	F	W	49	51	[6]UE	R521G	[9]fALS	Definite ([11]U+[12]L 3 reg)	frozen	motor cortex & lumbar cord	DN
A2	F	W	42	52	[7]LUE	H517Q	fALS	Definite (U+L 3 reg)	Paraffin; frozen	motor cortex & lumbar cord	16

A3	F	W	23	23	[8]B	Splice mutation IVS 13-2	fALS	Definite (U+L 3 reg)	Paraffin; frozen	motor cortex & lumbar cord	23
A4	F	W	11	17	LUE	P525L	[10]sALS	Definite (U+L 3 reg)	frozen	brain & lumbar cord	DN
A5	M	W	49	53	B	c.1408_ 1bpDEL(G) (codon 470 frame shift)	fALS	Probable Lab-Supported (U+L 1 reg or U 1 reg + L 2 + areas of [13]EMG)	frozen	brain & lumbar cord	DN

[1]M-Male; [2]F-Female; [3]W-White; [4]DN-Data not available; [5]N/A-Not Applicable; [6]UE-Upper Extremities; [7]LUE-Left Upper Extremeties; [8]B-Bulbar; [9]fALS-familial ALS; [10]sALS-sporadic ALS; [11]U-Upper Motor Neurons; [12]L-Lower Motor Neurons; [13]EMG-Electromyography; [14]central nervous system (CNS) region (specific regions of brain and spinal cord are listed for those cases where the information was available).

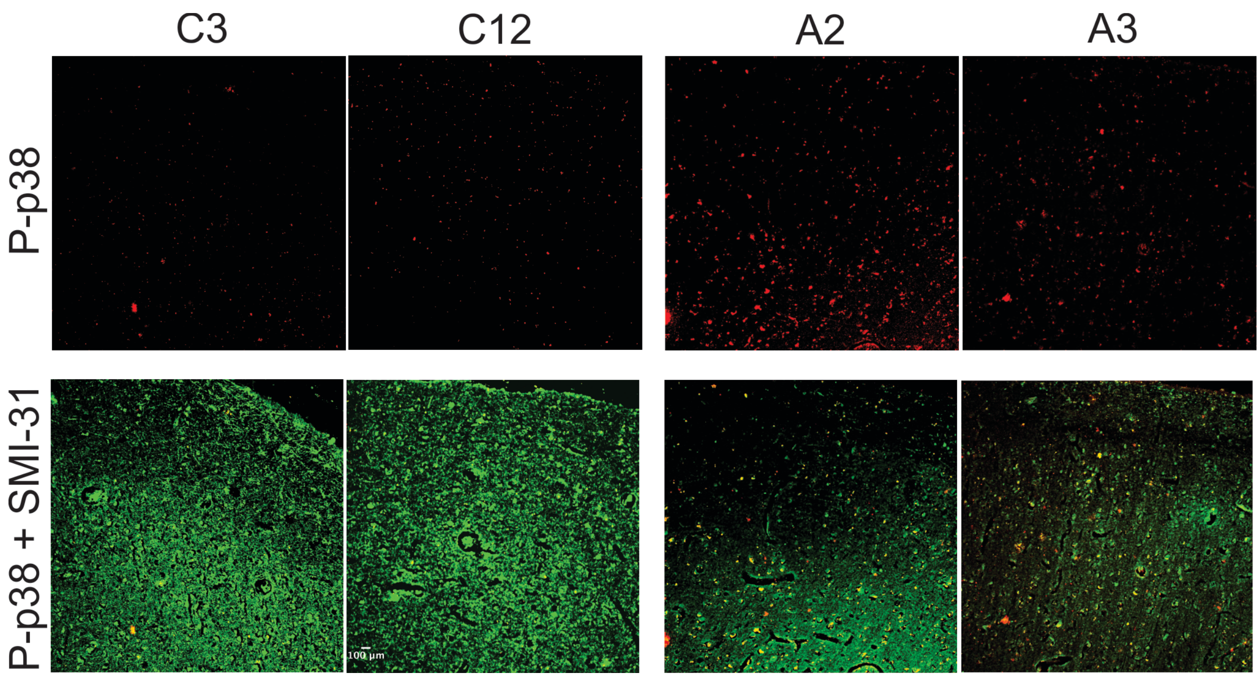

Supplemental Figure S3. Low magnification immunofluorescence images of post-mortem human CNS tissues. Low magnification images corresponding to the samples shown in Figure 6 within the main text. Brain sections from paraffin-embedded tissue samples obtained from two control (C3 and C12) and two ALS-FUS (A2 and A3) cases were probed with antibodies against phosphorylated, catalytically active p38 (P-p38; red) and phosphorylated neurofilament H (SMI31; green) as a marker of neuroaxonal integrity. Scale bar represents 100 µm.

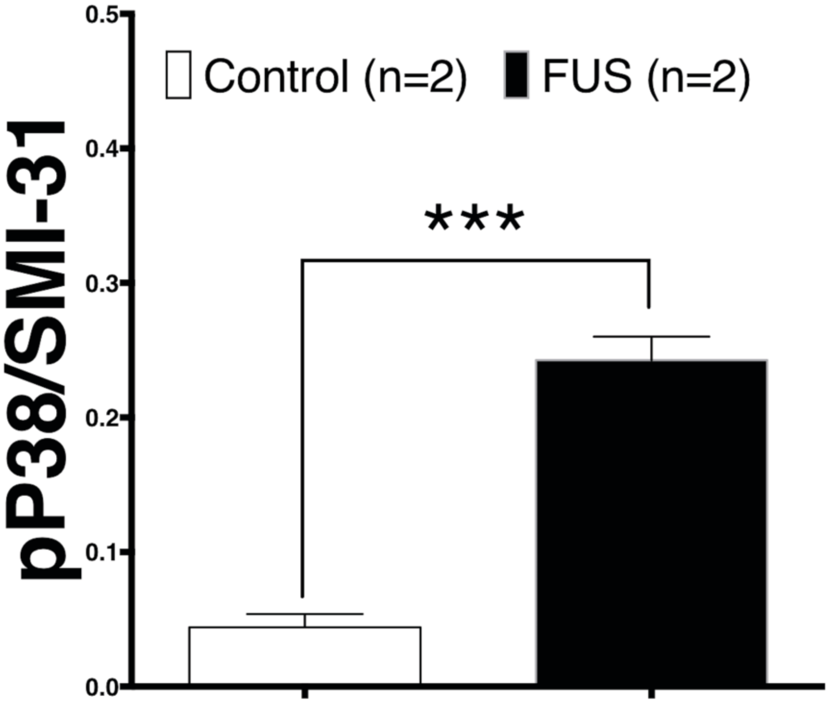

Supplemental Figure S4. Quantification of p-p38/SMI31 in human post-mortem motor cortex tissues. Quantification of images shown in Figure 6 in the main text and Supplemental Figure S3. Images corresponding to five regions of interest (ROIs) per case were acquired from motor cortex using the same settings. Images gathered from each channel (647nm for P-p38 and 488nm for SMI-31 antibodies, respectively) were processed and analyzed as described in the Methods. Statistical analysis (unpaired student t-test) was determined with Graph Pad Prism 6 ($p<0.0001$).
